# Radical cystectomy over the age of 75 is safe and increases survival

**DOI:** 10.1186/1471-2318-12-18

**Published:** 2012-04-30

**Authors:** Stavros I Tyritzis, Ioannis Anastasiou, Konstantinos G Stravodimos, Aristeides Alevizopoulos, Anastasios Kollias, Antonios Balangas, Ioannis Katafigiotis, Ioannis Leotsakos, Dionysios Mitropoulos, Constantinos A Constantinides

**Affiliations:** 1 Department of Urology, Athens University Medical School, LAIKO Hospital, Athens, Greece; 2 Department of Urology, Ammerland Clinics, Westerstede, Germany; 3 Department of Urology, Athens University Medical School, LAIKO Hospital, 17 Agiou Thoma str., Athens 11527, Greece

**Keywords:** Radical cystectomy, Octogenarians, Complications, Survival, Clavien classification system, Charlson comorbidity index

## Abstract

**Background:**

Radical cystectomy (RC) is probably underused in elderly patients due to a potential increased postoperative complication risk, as reflected by their considerable comorbidities. Our objective was to estimate the overall complication rate and investigate a potential benefit to patients over the age of 75 subjected to RC in terms of disease-free survival.

**Methods:**

A total of 81 patients, 61 men and 20 women, from two urological departments, with a mean age of 79.2 ± 3.7 years, participated in the study. The mean follow-up period was 2.6 ± 1.6 years. All patients underwent RC with pelvic lymphadenectomy. An ileal conduit, an orthotopic ileal neobladder and cutaneous ureterostomies were formed in 48.1%, 6.2% and 45.7% of the patients, respectively. The perioperative and 90-day postoperative complications were recorded and classified according to the modified Clavien classification system. Survival plots were created based on the oncological outcome and several study parameters.

**Results:**

The perioperative morbidity rate was 43.2%; the 90-day morbidity rate was 37%, while the 30-day, 90-day and overall mortality rates were 3.7%, 3.7% and 21%, respectively. Overall mortality rates were recorded at the final year of data gathering (2009). Increased age, increased body mass index (BMI), longer hospitalization and age-adjusted Charlson comorbidity index (ACCI) more than six, were associated with greater hazard for 90-day morbidity. The cumulative mortality / metastasis-free rates for one, two, three and five years were 88.7%, 77.5%, 70.4%, and 62.3%, respectively. Tumour stage and positive nodes were prognostic predictors for oncological outcome.

**Conclusions:**

RC in patients over 75 is justified and feasible, due to acceptable complication rates and high 5-year cancer-specific survival, which support an aggressive approach. Prospective studies are needed for the verification of the above results.

## Background

Muscle-invasive bladder cancer incidence increases with age and peaks in the octogenarians [1,2]. Even though radical cystectomy (RC) is the gold standard for this disease [3], it is underused and possibly inappropriately denied, because the majority of these patients are either unfit for surgery or have considerable comorbidities. This selection bias might preclude a potential effective management in the older patients [4]. The above argument is further strengthened by a recent report concluding that bladder-sparing protocols in octogenarians with > pT2 disease and American Society of Anesthesiologists (ASA) score 3–4 has a very bad prognosis [5].

Increased surgical experience and improvements in surgical techniques are constantly expanding the indications for aggressive treatment. This fact provided the rationale for our study, which attempted to investigate the feasibility of RC in these high-risk patients and establish a potential benefit on cancer-specific survival in the long term.

## Methods

We reviewed the files of 81 patients, 61 men and 20 women, with a mean age of 79.2 years (SD = 3.7 years) (range 75–95 years). All patients, after providing informed consent, underwent RC and pelvic lymphadenectomy from 2000 through 2009. Ethical approval was not required due to the retrospective nature of the study, as stated by our institutions scientific committee. The clinical and demographic characteristics of the patients are shown in Table 1. The indications for RC were muscle invasive disease, non muscle invasive disease refractory to intravesical chemotherapy and/or immunotherapy and palliation treatment for uncontrolled haemorrhage (in 11 out of 81 patients). No patient had distant metastasis at the time of the operation. No patient received neo-adjuvant chemotherapy or radiotherapy, while 12 (14.8%) and 3 patients (3.7%) received adjuvant chemotherapy and external beam radiotherapy, respectively.

**Table 1:** Demographic and clinical characteristics of the study group

	**N**	**%**
**Gender**		
Men	61	75.3
Women	20	24.7
**Age (years) , mean (SD)**	79.2(3.7)	
Age (years)*		
<77	23	28.4
77-80	31	38.3
>80	27	33.3
**BMI, mean (SD)**	25.5(3.2)	
**Urinary diversion**		
Ileal conduit	39	48.1
S-pouch	5	6.2
Cutaneous ureterostomy	37	45.7
**pT (TNM system)**		
pTis	12	14.8
pT1 to pT2b	28	34.6
pT3a to pT3b	29	35.8
pT4a to pT4b	12	14.8
**Nodes (TNM system)**		
0	60	74.1
1-2	21	25.9
**Hospital stay (days), mean (SD)**	13.0 (7.3)	
**Charlson score**		
≤6	69	85.2
>6	12	14.8

*categorized according to tertiles.

BMI: Body mass index.

SD: Standard deviation.

Perioperative complications were defined as complications occurring within the first 10 postoperative days. 90-day morbidity and mortality were also recorded. The Martin criteria for standardized reporting of complications were used [6] (Table 2). The complications were classified according to the modified Clavien classification system [7] (Table 3). Follow-up of the patients was performed by scheduled hospital visits and telephone interviews.

**Table 2:** Martin criteria for standardized complication reporting

**Criteria**	**Requirement**
Method of accruing data defined	Prospective or retrospective accrual of data are indicated.
Duration of follow-up indicated	Report clarifies the time period of postoperative accrual
	of complications such as 30 days or same hospitalization.
Outpatient information included	Study indicates that complications first identified following
	discharge are included in the analysis.
Definitions of complications provided	Article defines at least one complication with specific inclusion criteria.
Mortality rate and causes of death listed	The number of patients who died in the postoperative period of study
	are recorded together with cause of death.
Morbidity rate and total complications indicated	The number of patients with any complication and the total number
	of complications are recorded.
Procedure-specific complications included	
Severity grade utilized	Any grading system designed to clarify severity of complications
	including “major and minor” is reported.
Length-of-stay data	Median or mean length of stay indicated in the study.
Risk factors included in the analysis	Evidence of risk stratification and method used indicated by study.

**Table 3:** Clavien classification of perioperative and 90-day morbidity and mortality

**CLAVIEN grade**		**N**	**%**		**N**	**%**
**No complication**		**29**	**35.8**			
	**Perioperative morbidity (0-10d)**	**35**	**43.2**	**90-day morbidity**	**30**	**37**
**1**		**11**	**13.6**		**15**	**18.6**
Any deviation from the normal postoperative course without the need for pharmacological treatment or surgical, endoscopic, and radiological interventions. Allowed therapeutic regimens are: drugs as antiemetics, antipyretics, analgetics, diuretics, electrolytes, and physiotherapy. This grade also includes wound infections opened at the bedside.	Hyperemesis	1	1.2	Pneumonia	2	2.4
	Fever	7	8.4	Psoas abscess	1	1.2
	Ileus	1	1.2	Wound infection	11	13.6
	Pneumonia	1	1.2	Lympocele	1	1.2
	Urine leakage	1	1.2			
**2**		**22**	**27.2**		**4**	**4.8**
Requiring pharmacological treatment with drugs other than such allowed for grade I complications. Blood transfusions and total parenteral nutrition are also included.	Blood transfusion	21	25.9	Wound dehiscense	1	1.2
	UTI	1	1.2	UTI	3	3.6
**3**		**1**	**1.2**		**6**	**7.2**
Requiring surgical, endoscopic or radiological intervention						
**3a**	**-**	**-**	**0**		**2**	**2.4**
Intervention not under general anesthesia	-	-	0	Cardiac arrythmia-pacemaker	1	1.2
				Obstructive nephropathy	1	1.2
**3b**		**1**	**1.2**		**4**	**4.8**
Intervention under general anesthesia	Rectal injury, re-operation, colostomy	1	1.2	Evisceration, re-operation	1	1.2
				Stricture ileus, re-operation	2	2.4
				Incisional hernia	1	1.2
**4**		**1**	**1.2**		**2**	**2.4**
Life-threatening complication (including CNS complications) requiring IC/ICU management	Sepsis	1	1.2	Myocardial Infarction	1	1.2
				Sepsis	1	1.2
**5**	**-**	**-**	**0**		**3**	**3.7**
Death of a patient				**Death**	**3**	**3.7**
				*30-day* (sepsis, pneumonia)	2	2.4
				*60-day* (evisceration, re-operation	1	1.2
				*90-day*	0	0

### Statistical analysis

Quantitative variables are expressed as mean (±SD) or as median values (interquartile range). Qualitative variables are expressed as absolute and relative frequencies. Life table analyses were used to calculate cumulative survival rate (standard errors) for specific time intervals. The prognostic value of each variable was first assessed by univariate Cox regression analysis. Variables that showed significant association with the outcome were included in the multivariate Cox proportional-hazard model in a stepwise method, in order to determine the independent predictors for morbidity and oncological outcome. The assumption of proportional hazards was evaluated by testing for interaction with a continuous time variable. Kaplan – Meier survival estimates for soft and hard events were graphed over the follow-up period. All reported p values are two-tailed. Statistical significance was set at p < 0.05 and analyses were conducted using the SPSS statistical software (version 17.0).

## Results

**Figure 1:**
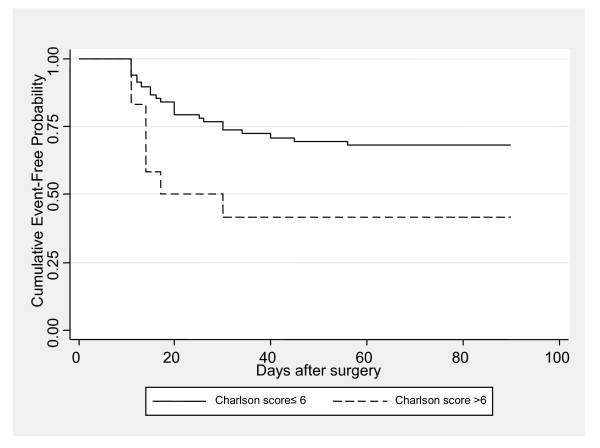
Kaplan-Meier curves for 90-day morbidity stratified by age-adjusted Charlson comorbidity index (p = 0.037).

The mean follow-up period was 2.6 ± 1.6 years with median equal to 2.4 years (interquartile range from 1.2 to 3.9 years). During the follow-up period, the perioperative morbidity rate was 43.2% (N = 35), 90-day morbidity rate was 35.8% (N = 29), the perioperative and overall mortality rates were 3.7% and 21.0%, respectively, while 6 patients had metastasis (7.4%). In salvage RC, perioperative morbidity rate was 45.4%, 90-day morbidity rate reached 36.4%, while perioperative mortality was 9%. Positive nodes were found in 25.9% of the patients. The mean length of hospitalization was 13.0 ± 7.3 days and 14.8% of the patients had an age-adjusted Charlson comorbidity index (ACCI) more than six. Table 4 presents the results of univariate analysis for the association of study parameters with perioperative morbidity. Perioperative morbidity rate was not significantly different when adjusted to the clinical and demographic characteristics of the patients. Univariate Cox regression analysis for 90-day morbidity (Table 5) revealed that increased age, increased body mass index (BMI), hospital stay and ACCI more than six, were associated with greater hazard for 90-day morbidity. When multiple Cox regression analysis with stepwise-forward approach was applied (Table 6), it was found that age, BMI, hospital stay and ACCI were independently associated with morbidity. Specifically, for one day increase in hospital stay, the morbidity hazard increases by 6%, while for one unit increase in BMI, the morbidity hazard increases by 18%. Furthermore, patients aged 77 to 80 years and those aged more than 80 years had greater morbidity hazard compared to those aged less than 77 years with adjusted hazard ratios equal to 4.92 and 5.86, respectively. Additionally, subjects with ACCI more than six had 2.51 times greater morbidity hazard compared to those with ACCI equal or less than six. Kaplan Meier morbidity estimations according to ACCI are presented in Figure 1.

**Table 4:** Demographic and clinical characteristics of the study group associated with perioperative morbidity

	**Perioperative morbidity (10 days)**				**Px² test**
	**No**		**Yes**		
	**N**	**%**	**N**	**%**	
**Gender**					
Male	36	59.0	25	41.0	0.752
Female	11	55.0	9	45.0	
**Age (years) , mean (SD)**	79.3(4.0)		78.9(3.2)		0.650**
**Age (years)**					
<77	14	60.9	9	39.1	0.895
77-80	17	54.8	14	45.2	
>80	16	59.3	11	40.7	
**BMI, mean (SD)**	25.6(2.9)		25.5(3.7)		0.873**
**Urinary diversion**					
Ileal conduit	22	56.4	17	43.6	0.724*
S-pouch	4	80.0	1	20.0	
Cutaneous ureterostomy	21	56.8	16	43.2	
**pT (TNM system)**					
pTis	3	25.0	9	75.0	0.070
pT1 to pT2b	17	60.7	11	39.3	
pT3a to pT3b	18	62.1	11	37.9	
pT4a to pT4b	9	75.0	3	25.0	
**Nodes (TNM system)**					
0	34	56.7	26	43.3	0.675
1-2	13	61.9	8	38.1	
**Hospital stay, mean (SD)**	13.7(8.4)		12.1(5.4)		0.334**
**Charlson score**					
≤6	39	56.5	30	43.5	0.511
>6	8	66.7	4	33.3	

*Indicates reference category.

**Hazard ratio cannot be calculated because of no distribution.

**Table 5:** Univariate and multiple Cox proportional hazards models for the prediction of 90-day morbidity

	**Crude**		**Adjusted**	
	**HR (95% CI) ‡**	**P**	**HR (95% CI)**	**P**
**Gender**				
Male	1.00*			
Female	0.98 (0.42 - 2.28)	0.955		
**Age (years), mean ± SD**	1.08 (0.99 - 1.17)	0.079		
**Age (years)**				
<77	1.00*		1.00*	
77-80	4.12 (1.18 - 14.33)	0.026	4.92 (1.35 - 17.95)	0.016
>80	4.04 (1.14 - 14.31)	0.031	5.86 (1.47 - 23.42)	0.012
**BMI, mean ± SD**	1.15 (1.04 - 1.27)	0.008	1.18 (1.04 - 1.34)	0.011
**Urinary diversion**				
Ileal conduit	1.00*			
S-pouch	**			
Cutaneous ureterostomy	1.18 (0.57 - 2.44)	0.658		
**pT (TNM system)**				
pTis	1.00*			
pT1 to pT2b	0.51 (0.18 - 1.42)	0.197		
pT3a to pT3b	0.62 (0.23 - 1.72)	0.362		
pT4a to pT4b	0.56 (0.16 - 2)	0.377		
**Nodes (TNM system)**				
0	1.00*			
1-2	1.2 (0.53 - 2.7)	0.665		
**Hospital stay, mean ± SD**	1.05 (1.02 - 1.09)	0.002	1.06 (1.02 - 1.09)	0.002
**Charlson score**				
≤6	1.00*		1.00*	
>6	2.43 (1.03 - 5.7)	0.042	2.51 (1.06 - 5.94)	0.037

‡Hazard Ratio (95% Confidence Interval).

*Indicates reference category.

**Hazard ratio cannot be calculated because of no distribution.

**Table 6:** Univariate and multiple Cox proportional hazards models for the prediction of death/metastasis

	**Crude**		**Adjusted**	
	**HR (95% CI)‡**	**P**	**HR (95% CI)**	**P**
**Gender**				
Male	1.00*			
Female	0.41 (0.12 - 1.4)	0.155		
**Age (years), mean ± SD**	1 (0.89 - 1.13)	0.976		
**Age (years)**				
<77	1.00*			
77-80	1.57 (0.57 - 4.34)	0.382		
>80	1.06 (0.36 - 3.17)	0.910		
**BMI, mean ± SD**	0.96 (0.84 - 1.1)	0.563		
**Urinary diversion**				
Ileal conduit	1.00*	0.997		
S-pouch	**			
Cutaneous ureterostomy	1.03 (0.45 - 2.36)	0.944		
**pT (TNM system)**				
pTis	1.00*		1.00*	
pT1 to pT2b	3.11 (0.37 - 25.88)	0.293	2.87 (0.35 - 23.9)	0.329
pT3a to pT3b	5.5 (0.7 - 43.04)	0.105	5.99 (0.76 - 46.95)	0.088
pT4a to pT4b	9.11 (1.09 - 75.81)	0.041	9.19 (1.10 - 76.58)	0.040
**Nodes (TNM system)**				
0	1.00*		1.00*	
1-2	2.52 (1.10 – 5.75)	0.028	2.4 (1.05 - 5.49)	0.039
**Hospital stay. mean ± SD**	0.99 (0.93 - 1.06)	0.777		

**Figure 2:**
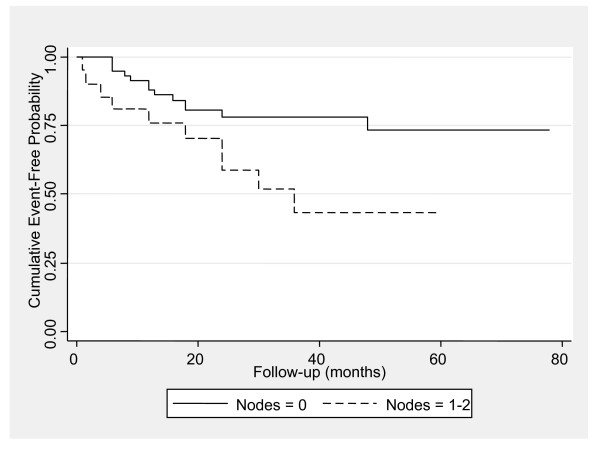
Kaplan-Meier curves for event-free probability stratified by nodal status. (p = 0.039).

**Figure 3:**
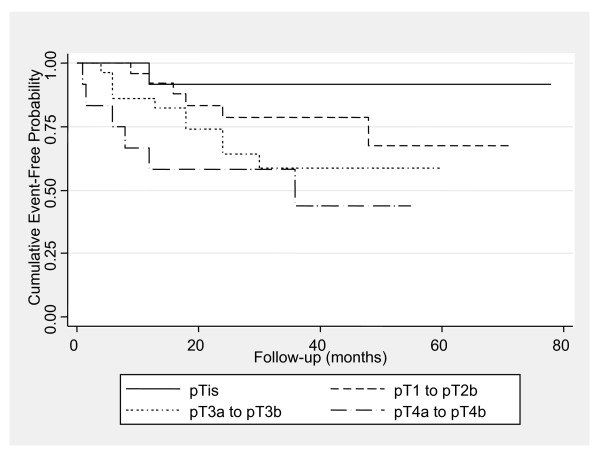
**Kaplan-Meier curves for event-free probability stratified by tumour stage.** (pT1-pT2b: p = 0.329, pT3: p = 0.08, pT4: p = 0.04).

The cumulative mortality / metastasis-free rates for one, two, three and five years were 88.7% [Standard Error (SE) = 3.6%], 77.5% (SE = 4.8%), 70.4% (SE = 5.5%) and 62.3% (SE = 7.3%), respectively. Both univariate and multiple analysis concerning death or metastasis (Table 5) revealed that tumour stage and nodes were prognostic predictors for oncological outcome. Specifically, as resulted from multiple analysis, patients with tumour stage pT4a to pT4b had 9.19 times greater hazard for death or metastasis compared to those with in situ tumours. Moreover, patients with one or two positive nodes had 2.4 times greater hazard for death or metastasis. Kaplan Meier estimates for death or metastasis according to tumour stage and nodes are presented in Figures 2 and 3, respectively.

## Discussion

Octogenarians are per se, a high-risk group, due to their comorbidities. Using the age-adjusted Charlson comorbidity index, for each decade over the age of 40 years, one point is added to the total score, a fact that reflects the significance of age in the performance status of a patient [8]. Koppie et al recently suggested the association between ACCI and the clinical and oncological outcome after a RC [9]. Other authors also reported that postoperative morbidity is higher in patients with higher ASA score and more than 2 comorbidities [10]. The same conclusion was reached by a collaborative review, which however, stated that age alone does not preclude RC in the elderly [11].

Our results of perioperative mortality and 90-day morbidity are comparable to those of the above mentioned reviews, which range between 0-11% and as high as 64% [11], respectively, support the notion of an aggressive treatment offered to octogenarians. The 90-day instead of the 30-day or the 60-day morbidity was selected because it is already established as a more potent and realistic description tool of the postoperative risks of the RC morbidity, as several reports are suggesting [11,12]. The 90-day morbidity along with the use of the Clavien classification system, give a distinct advantage in our study in terms of complication reporting quality [11-13].

The incidence of wound complications was surprisingly high in our series (11%) and it could be attributed to poor healing capacity and poor patient hygiene. In order to avoid this kind of complication, we now routinely use tension sutures along with Nylon sutures or clips for wound closure at the end of the operation, which are removed approximately one month later.

The complication rates did not vary between salvage and primary RC. The main difference was that perioperative mortality was significantly higher in the salvage RC (9% vs. 3.7%), which was somewhat expected, due to the nature of the disease and the performance status of the patient at the time of the operation. The above observation is in accordance to other authors [14,15]. Furthermore, obesity and longer hospital stay have been identified as risk factors for higher postoperative morbidity.

The selected type of urinary diversion does not seem to alter the morbidity/mortality outcome, as shown in other studies as well [11,16]. It is however important to notice, that high-volume centers, performing > 50 RC’s per year should be involved in these cases, because the surgical experience in these centers is more advanced and is associated with improved postoperative outcomes, including decreased mortality, shorter length of hospital stay and lower rehospitalization rates [11,17].

Cancer-specific survival is another major concern when the decision to perform RC in an octogenarian should be taken, as the reported results are rather controversial. Two retrospective studies, reviewing a total of approximately 1120 patients have reported unfavourable oncological outcomes in elderly patients [18,19]. In the first study, the 5-year was 28.1%, while in the second the 3-year and 7-year cancer-specific survival rates were between 70% and 55.2%, respectively. On the other hand, Chamie et al and Hollenbeck et al provided some evidence that RC has some benefit in the elderly, when compared to watchful waiting, or radiotherapy and chemotherapy [4,20]. Chamie and colleaques also stated that pelvic lymphadenectomy should be performed in order for RC to show any survival advantage in the octogenarians [20]. Based on our results, we advocate RC and lymphadenectomy in the elderly patients, since the 5-year survival rate in our series was significantly high (62.3%) and tumour stage and node status were prognostic predictors for the oncological outcome. Clinical tumour stage and grade remain the best predictors of cancer specific survival [21]. Therefore, it is imperative for the RC candidates to undergo a meticulous clinical and imaging examination.

## Conclusions

Age should not hamper RC, particularly when it takes place in specialized, high-volume centers. The reported complication rates of this particular group of patients, especially when standardized with validated methodologies, are acceptable. Strict selection of non obese patients, with non metastatic disease and an ACCI less than six, may result in a better postoperative outcome.

## Competing interests

The authors declare that they have no competing interests.

## Authors’ contributions

IA performed surgical procedures, participated in the study concept and design and critically revised the manuscript. SIT conceived the study, performed statistical analysis, drafted the manuscript, analyzed and interpreted the data. KGS performed surgical procedures, assisted in the study concept and design and revised the manuscript. AA participated in the acquisition of data. AK performed surgical procedures and provided patient data. AB participated in the acquisition of data. VM participated in the acquisition of data. DM performed surgical procedures, assisted in the study concept and design and revised the manuscript. CAC performed surgical procedures, assisted in the study concept and design and revised the manuscript. All authors read and approved the final manuscript.

## Pre-publication history

The pre-publication history for this paper can be accessed here:

http://www.biomedcentral.com/1471-2318/12/18/prepub
